# Effect of androgen deprivation therapy on serum levels of sclerostin, Dickkopf-1, and osteoprotegerin: a cross-sectional and longitudinal analysis

**DOI:** 10.1038/s41598-021-94090-y

**Published:** 2021-07-21

**Authors:** Alice Wang, Nishi Karunasinghe, Lindsay D. Plank, Shuotun Zhu, Sue Osborne, Charis Brown, Karen Bishop, Tiffany Schwass, Sofian Tijono, Michael Holmes, Jonathan Masters, Roger Huang, Christine Keven, Lynnette R. Ferguson, Ross Lawrenson

**Affiliations:** 1 Discipline of Nutrition and Dietetics, University of Auckland, Auckland, New Zealand; 2 Auckland Cancer Society Research Centre, University of Auckland, Auckland, New Zealand; 3 Department of Surgery, University of Auckland, Auckland, New Zealand; 4 Urology Department, North Shore Hospital, Auckland, New Zealand; 5 The Medical Research Centre, University of Waikato, Waikato, New Zealand; 6 Urology Department, Waikato Hospital, Hamilton, New Zealand; 7 Urology Department, Whangarei Hospital, Whangarei, New Zealand; 8 Radiation Oncology Department, Waikato Hospital, Hamilton, New Zealand; 9 The Liggins Institute, University of Auckland, Auckland, New Zealand

**Keywords:** Endocrinology, Oncology, Urology

## Abstract

Androgen deprivation therapy (ADT) for men with prostate cancer (PCa) results in accelerated bone loss and increased risk of bone fracture. The aim of the present study was to evaluate serum bone markers—sclerostin, Dickkopf-1 (DKK-1) and osteoprotegerin (OPG), in a cohort of 88 PCa patients without known bone metastases, managed with and without ADT, and to analyse their relationship with bone mineral density (BMD) and sex steroids. The cross-sectional analysis between acute-, chronic- and former-ADT groups and PCa controls showed that sclerostin and OPG levels significantly differed between them (*p* = 0.029 and *p* = 0.032). Groups contributing to these significant changes were recorded. There were no significant differences in serum DKK-1 levels across the four groups (*p* = 0.683). In the longitudinal analysis, significant % decreases within groups were seen for DKK-1 [chronic-ADT (− 10.06%, *p* = 0.0057), former-ADT (− 12.77%, *p* = 0.0239), and in PCa controls group (− 16.73, *p* = 0.0022); and OPG levels in chronic ADT (− 8.28%, *p* = 0.003) and PCa controls group (− 12.82%, *p* = 0.017)]. However, % changes in sclerostin, DKK-1, and OPG did not differ significantly over 6-months across the evaluated groups. Sclerostin levels showed significant positive correlations with BMD at baseline in the ADT group, while in PCa controls this correlation existed at both baseline and 6-month time points. Sclerostin correlated negatively with testosterone in former ADT users and in PCa controls. Possible prognostic features denoted by parallel increases in sclerostin and BMD are discussed.

## Introduction

Androgen deprivation therapy (ADT) is the mainstay of treatment for men with metastatic prostate cancer (PCa), and it is also commonly used in earlier stages of disease, particularly in combination with radiation therapy in high risk localised or locally advanced disease^1,2^. Despite its effectiveness in treating PCa, ADT comes with a range of side effects that negatively impact patient’s quality of life. In particular, it is associated with accelerated bone loss and increased risk of bone fracture^3^.

During normal bone remodelling, bone resorption and formation are balanced due to the activity of osteoclasts and osteoblasts. Osteoporosis is characterised by an imbalance between bone formation and bone resorption. The receptor activator of NF-kappa B ligand (RANKL)/RANK/osteoprotegerin (OPG) signaling pathway plays a crucial role in regulating bone resorption^4^. Osteocytes, the terminally differentiated osteoblasts embedded in the mineralized bone matrix, are the most abundant bone cells (> 90% of bone cells)^5^. They play an important role in bone remodelling by controlling activities of osteoclasts and osteoblasts through the production of RANKL and sclerostin. Recently, it has been implicated that osteocytes are the major source of RANKL^6−8^. By binding to its receptor, RANK, on osteoclast precursors, RANKL controls the differentiation, proliferation, and survival of osteoclasts. OPG is secreted by osteoblasts, and it binds to RANKL as a decoy receptor, and blocks its interaction with RANK, thus blocking osteoclast formation and bone resorption^4^.

Activation of the Wnt signaling pathway stimulates differentiation, proliferation and survival of osteoblasts, which results in increased bone formation^9,10^. Sclerostin, encoded by the *SOST* gene, is a 190-amino acid glycoprotein secreted mainly by osteocytes, and it acts as a negative regulator of bone formation through inhibiting the Wnt signaling pathway^11^. The function of sclerostin in bone metabolism is illustrated by two rare high bone mass disorders, Sclerosteosis and Van Buchem disease, characterised by deficiency or impaired sclerostin production. Sclerosteosis results from a loss-of-function mutation in the sclerostin gene, and Van Buchem disease is caused by deletion of a large regulatory element of the gene^12,13^. Dickkopf-1 (DKK-1) is another potent inhibitor of Wnt signaling. Sclerostin and DKK-1 bind to Wnt co-receptors, LRP5/6, and block Wnt signaling, leading to inhibition of differentiation and proliferation of osteoblasts, and resulting in inhibition of bone formation^10,14^.

The effect of ADT on serum levels of sclerostin, DKK-1 and OPG are not well studied. The current study aimed to evaluate cross-sectional and longitudinal variability of serum sclerostin, DKK-1 and OPG levels in a cohort of PCa patients managed with and without ADT. In addition, we aimed to analyse the relationship of these bone markers with BMD and sex steroids.

## Results

### Baseline cross-sectional characteristics between study groups

There were no significant differences in age, weight, height, BMI, parathyroid hormone and 25-Hydroxyvitamin D levels across the four groups (Table 1). The total body BMD differ significantly across the groups (*p* = 0.032). Total body BMD was significantly higher in former ADT users and PCa controls as compared to chronic ADT users (1.188 g/cm^2^ and 1.214 g/cm^2^ vs 1.096 g/cm^2^, *p* = 0.044 and *p* = 0.004, respectively). There were significant differences in serum sclerostin levels across the groups (*p* = 0.029) (Table 2). Serum sclerostin levels were significantly higher in men receiving acute or chronic ADT than PCa controls (3099 pg/mL and 2971.95 pg/mL vs. 2621.55 pg/mL, *p* = 0.007 and *p* = 0.033, respectively). Acute ADT users also had significantly higher levels of serum sclerostin than former ADT users (3099 pg/mL vs 2309.84 pg/mL, *p* = 0.031). There were no significant differences in serum DKK-1 levels across the four groups (*p* = 0.683). The baseline levels of OPG differed significantly across the groups (*p* = 0.032). Serum OPG levels were significantly higher in chronic ADT users and former ADT users as compared to PCa controls (404.56 pg/mL and 426.51 pg/mL vs 329.90 pg/mL, *p* = 0.012 and *p* = 0.013, respectively) (Table 2).

**Table 1:** Baseline characteristics, BMD and hormone levels.

	Acute ADT (n = 7)	Chronic ADT (n = 30)	Former ADT users (n = 21)	PCa Controls (n = 30)	*p* value
Age	71.6 (63.22–77.75)	71.15 (67.98–73.97)	74.50 (70.50–77.23)	69.70 (68.45–72.45)	0.243
Weight	91.1 (81.81–104.45.0)	87.20 (83.48–96.76)	79.50 (77.38–90.84)	85.25 (80.42–91.50)	0.313
Height	169 (167.54–174.89)	175.25 (171.07–176.78)	175.20 (170.71–176.63)	176.50 (173.38–178.80)	0.157
BMI	32.50 (28.31–35.17)	29.30 (27.91–31.48)	28.00 (25.96–29.73)	27.65 (26.06–29.36)	0.085
**BMD**					
Lumbar spine (g/cm^2^)	1.194 (1.158–1.314)	1.165 (1.093–1.247)	1.312 (1.184–1.410)	1.307 (1.229–1.375)	0.094
Femoral neck (g/cm^2^)	0.949 (0.745–1.083)	0.864 (0.836–0.939)	0.858 (0.826–0.973)	0.946 (0.905–0.990)	0.242
One-third distal radius (g/cm^2^)	0.975 (0.806–1.061)	0.918 (0.882–0.960)	0.950 (0.913–1.003)	0.990 (0.944–1.028)	0.098
Ultradistal forearm (g/cm^2^)	0.471 (0.411–0.526)	0.446 (0.415–0.465)	0.477 (0.442–0.508)	0.483 (0.445–0.509)	0.17
Total body (g/cm^2^)	1.171 (1.052–1.346)	1.096 (1.072–1.184)	1.188 (1.134–1.239)	1.214 (1.166–1.262)	0.032
**Hormones**					
Testosterone (ng/mL)	0.048 (− 0.769 to 2.049)	0.063 (0.047–0.167)	3.780 (2.448–5.087)	4.335 (3.802–4.870)	< 0.0001
Estradiol (pg/mL)	5.0 (− 20.31 to 67.39)	5.00 (4.39–7.56)	24.40 (16.53–28.99)	23.24 (20.92–26.60)	< 0.0001
Parathyroid hormone (pg/mL)	39.14 (31.08–56.48)	42.93 (36.56–48.47)	41.20 (37.25–48.98)	39.91 (37.98–49.38)	0.995
25-Hydroxyvitamin D (ng/mL)	24.10 (18.43–32.15)	29.91 (26.41–35.39)	29.99 (25.64–35.85)	32.55 (29.00–36.49)	0.305

Results for continuous variables are presented as median (95% CI). Testosterone: 1 ng/mL = 3.47 nmol/L; Estradiol: 1 pg/mL = 3.671 pmol/L; Parathyroid hormone: 1 pg/mL = 0.106 pmol/L; 25-hydroxyvitamin D: 1 ng/mL = 2.496 nmol/L.

**Table 2:** Baseline serum levels of bone markers.

Baseline	Acute ADT (n = 7)	Chronic ADT (n = 30)	Former ADT users (n = 21)	PCa Controls (n = 30)	*p* value
DKK-1 (pg/mL)	1288.09 (881.57–1980.65)	1161.04 (1143.91–1442.62)	1332.65 (1186.30–1498.84)	1237.03 (1147.87–1437.50)	0.683
Sclerostin (pg/mL)	3099.91 (1814.84–5849.45)	2971.95 (2596.11–3274.92)	2309.84 (2138.82–3093.34)	2621.55 (2196.47–2688.09)	0.029
OPG (pg/mL)	350.23 (159.91–730.62)	404.56 (384.99–475.76)	426.51 (373.30–527.94)	329.90 (306.14–429.88)	0.032

6 months	Acute ADT (n = 6)	Chronic ADT (n = 24)	Former ADT users (n = 16)	PCa Controls (n = 26)	
DKK-1 (pg/mL)	1072.35 (741.22–1717.95)	1004.37 (996.29–1285.94)	1216.66 (1118.74–1364.56)	1011.25 (942.17–1136.37)	0.077
Sclerostin (pg/mL)	2267.46 (483.37–6441.37)	2942.04 (2784.74–3499)	2606.02 (2359.12–3335.16)	2328.44 (2155.13–2778.16)	0.045
OPG (pg/mL)	350.22 (136.03–687.46)	402.20 (367.14–479.43)	373.43 (345.60–522.62)	290.49 (257.98–380.31)	0.006

Results are presented as median (95% CI). *DKK1* Dickkopf-1, *OPG* osteoprotegerin.

### Correlation of baseline bone markers with age and BMI

Baseline serum sclerostin levels were positively correlated with age in acute and chronic ADT users (correlation coefficient r_s_ = 0.498, *p* = 0.002). There was a significant positive correlation between sclerostin levels and BMI in PCa controls (r_s_ = 0.442, *p* = 0.015). A negative correlation between serum DKK-1 and age was observed in former ADT users (r_s_ = −0.513, *p* = 0.017). Serum OPG was positively correlated with age in patients receiving ADT (r_s_ = 0.511, *p* = 0.001), and former ADT users (r_s_ = 0.568, *p* = 0.009) (Table 3).

**Table 3:** Correlation of serum sclerostin, DKK-1, OPG, age and BMI.

	Sclerostin		DKK-1		OPG	
	r_s_	*p* value	r_s_	*p* value	r_s_	*p* value
**Acute and chronic ADT users (n = 37)**						
Age	0.498	0.002	− 0.265	0.118	0.511	0.001
BMI	0.010	0.956	0.097	0.572	− 0.053	0.758
**Former ADT users (n = 21)**						
Age	0.258	0.258	− 0.513	0.017	0.568	0.009
BMI	0.026	0.911	0.464	0.034	− 0.327	0.160
**PCa controls (n = 30)**						
Age	0.138	0.468	0.010	0.958	0.316	0.089
BMI	0.442	0.015	0.120	0.527	0.326	0.079

*DKK1* Dickkopf-1, *OPG* osteoprotegerin.

### Longitudinal changes in serum levels of sclerostin, DKK-1, and OPG

**Figure 1:**
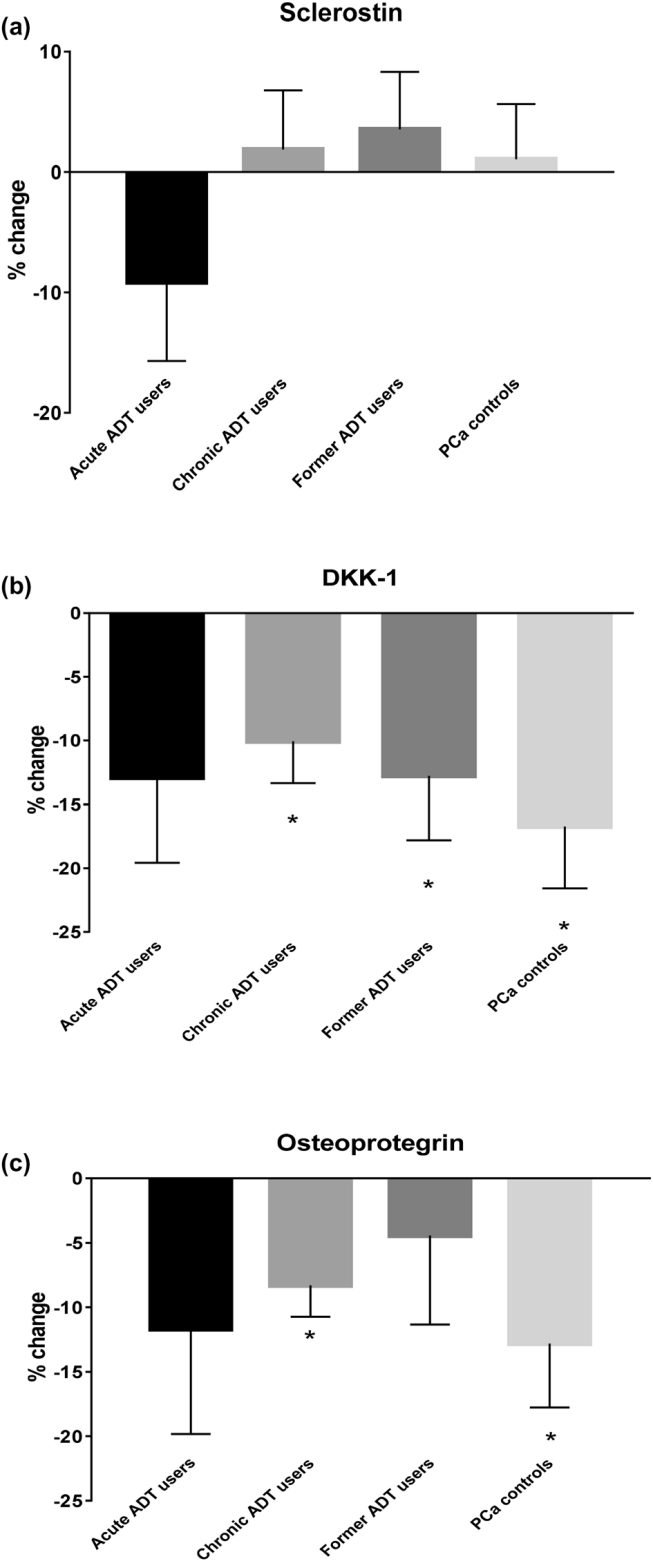
Least squares mean percentage changes from baseline to 6 months in serum (**a**) sclerostin, (**b**) DKK-1, and (**c**) OPG. Acute ADT users n = 6, chronic ADT users n = 24, Former ADT users n = 16, and PCa controls n = 26. *Significantly changed from baseline, *p* < 0.05.

There were no significant changes from baseline to 6 months in serum sclerostin levels in any of the four groups. The least squares (LS) mean changes in serum sclerostin were − 9.15%, + 1.87%, + 3.54%, and + 1.06% among acute ADT users, chronic ADT users, former ADT users, and PCa controls, respectively (Fig. 1a). Serum DKK-1 significantly decreased from baseline to 6 months in chronic ADT users (− 10.06%, *p* = 0.0057), former ADT users (− 12.77%, *p* = 0.0239), and PCa controls (− 16.73, *p* = 0.0022), but not in acute ADT users (12.91%, *p* = 0.11) (Fig. 1b).

A significant reduction from baseline to 6 months in serum OPG levels was observed in chronic ADT users (− 8.28%, *p* = 0.003) and PCa controls (− 12.82%, *p* = 0.017). Men on acute ADT and former ADT users also had a decreased OPG level, but the changes were not significant (− 11.69%, *p* = 0.21 and − 4.42%, *p* = 0.533, respectively) (Fig. 1c). The percentage change in serum sclerostin (*p* = 0.37), DKK-1 (*p* = 0.75) and OPG (*p* = 0.74) levels did not differ significantly across the groups (data not shown).

### Correlation of bone markers and BMD

The correlations between bone markers and BMD are shown in Table 4. Sclerostin levels were positively correlated with lumbar spine (r_s_ = 0.435, *p* = 0.009), ultradistal forearm (r_s_ = 0.336, *p* = 0.049) and total body (r_s_ = 0.526, *p* = 0.002) BMD in acute plus chronic ADT users at baseline. Significant positive correlations between serum sclerostin levels and lumbar spine (r_s_ = 0.580, *p* = 0.0008) and total body (r_s_ = 0.422, *p* = 0.02) BMD were also observed in PCa controls at baseline. This correlation with lumbar spine BMD remained significant in PCa controls even after 6 months (r_s_ = 0.601, *p* = 0.001). No significant correlation with BMD was observed in former ADT users at baseline.

**Table 4:** Correlation between bone markers and BMD.

	DKK-1		Sclerostin		OPG	
	r_s_	*p* value	r_s_	*p* value	r_s_	*p* value
**Acute and chronic ADT users**						
Baseline (n = 37)						
L2–L4 lumbar spine	− 0.195	0.262	0.435	0.009	0.146	0.404
Femoral neck	0.295	0.080	0.126	0.465	− 0.011	0.950
One-third distal radius	− 0.258	0.134	0.190	0.274	− 0.272	0.114
Ultradistal forearm	− 0.271	0.115	0.336	0.049	− 0.042	0.809
Total body	− 0.053	0.774	0.526	0.002	− 0.064	0.727
6 months (n = 30)						
L2–L4 lumbar spine	0.146	0.458	0.197	0.315	0.198	0.323
Femoral neck	0.166	0.398	− 0.180	0.360	− 0.160	0.426
One-third distal radius	− 0.089	0.654	0.008	0.967	− 0.159	0.429
Ultradistal forearm	− 0.028	0.888	0.067	0.734	− 0.059	0.769
Total body	0.218	0.295	0.259	0.211	− 0.147	0.492
**Former ADT users**						
Baseline (n = 21)						
L2–L4 lumbar spine	0.327	0.148	− 0.070	0.763	− 0.236	0.316
Femoral neck	0.317	0.173	− 0.299	0.200	− 0.318	0.185
One-third distal radius	− 0.326	0.149	− 0.211	0.360	− 0.013	0.957
Ultradistal forearm	0.016	0.947	− 0.164	0.477	− 0.199	0.400
Total body	0.226	0.325	− 0.248	0.278	− 0.296	0.205
6 months (n = 16)						
L2–L4 lumbar spine	0.325	0.237	− 0.404	0.136	− 0.029	0.920
Femoral neck	0.257	0.375	− 0.437	0.118	− 0.051	0.864
One-third distal radius	− 0.071	0.800	− 0.511	0.052	− 0.118	0.676
Ultradistal forearm	0.389	0.152	− 0.414	0.125	0.007	0.980
Total body	0.125	0.657	− 0.625	0.013	− 0.139	0.621
**PCa controls**						
Baseline (n = 30)						
L2–L4 lumbar spine	0.294	0.115	0.580	0.001	0.430	0.018
Femoral neck	0.226	0.230	0.230	0.222	0.237	0.207
One-third distal radius	0.110	0.564	0.211	0.263	0.091	0.631
Ultradistal forearm	− 0.098	0.606	0.269	0.151	0.238	0.206
Total body	0.153	0.420	0.422	0.020	0.352	0.056
6 months (n = 26)						
L2–L4 lumbar spine	0.378	0.062	0.601	0.001	0.396	0.050
Femoral neck	0.183	0.382	0.169	0.410	0.102	0.626
One-third distal radius	0.178	0.394	0.119	0.562	− 0.004	0.985
Ultradistal forearm	0.008	0.971	0.183	0.371	0.151	0.472
Total body	0.091	0.666	0.294	0.144	0.189	0.367

*DKK1* Dickkopf-1, *OPG* osteoprotegerin.

There was no significant correlation between DKK-1 and BMD in ADT users and PCa controls. In PCa controls, a significant correlation between OPG and lumbar spine BMD was observed at baseline (r_s_ = 0.430, *p* = 0.018) and after 6 months of follow-up (r_s_ = 0.396, *p* = 0.05).

### Correlation between sex steroids and bone markers

The correlations between sex steroids and bone markers are shown in Table 5. In PCa controls, sclerostin was negatively correlated with testosterone at both baseline (r_s_ = −0.413, *p* = 0.023) and at 6 months (r_s_ = −0.532, *p* = 0.005) but not with estradiol. No correlations were observed with these sex steroids at either time point among acute and chronic ADT users. A significant inverse correlation between serum sclerostin and testosterone was observed in the former ADT group at baseline (r_s_ = −0.588, *p* = 0.005), but the correlation did not persist after 6 months of follow-up.

**Table 5:** Correlations between bone markers and sex steroids.

	DKK-1		Sclerostin		OPG	
	r_s_	*p* value	r_s_	*p* value	r_s_	*p* value
**Acute and chronic ADT users**						
Baseline (n = 37)						
Testosterone	0.333	0.047	− 0.040	0.818	0.044	0.801
Estrogen	0.195	0.254	0.005	0.976	0.088	0.610
6 months (n = 30)						
Testosterone	0.096	0.627	− 0.265	0.173	− 0.078	0.698
Estrogen	− 0.180	0.360	− 0.173	0.378	0.094	0.641
**Former ADT user**						
Baseline (n = 21)						
Testosterone	− 0.152	0.511	− 0.588	0.005	− 0.341	0.141
Estrogen	− 0.090	0.697	− 0.393	0.078	− 0.205	0.387
6 months (n = 16)						
Testosterone	− 0.079	0.781	− 0.389	0.152	− 0.296	0.283
Estradiol	− 0.261	0.348	− 0.157	0.576	0.161	0.567
**PCa controls**						
Baseline (n = 30)						
Testosterone	0.077	0.687	− 0.413	0.023	− 0.178	0.346
Estrogen	0.220	0.242	0.012	0.951	− 0.006	0.977
6 months (n = 26)						
Testosterone	− 0.076	0.718	− 0.532	0.005	− 0.285	0.167
Estrogen	0.165	0.432	0.108	0.601	0.038	0.858

*DKK1* Dickkopf-1, *OPG* osteoprotegerin.

A positive correlation between serum DKK-1 and testosterone was observed in men receiving ADT at baseline (r_s_ = 0.333, *p* = 0.047). There were no correlations between serum OPG and either testosterone or estradiol in ADT users or PCa controls.

## Discussion

Among the four groups evaluated, the age, weight, height, BMI were comparable. Parathyroid hormone levels and vitamin D levels were also comparable between the study groups. However, the median parathyroid hormone levels recorded among our four groups (39.14–42.93 pg/mL equivalent to 4.14–4.55 pmol/L) were marginally higher than the levels (3.10 pmol/L) recorded for PCa patients with bone metastasis from USA with similar median age to our cohort^15^. Murray et al.^16^ record median plasma parathyroid hormone levels among PCa patients of similar age to our cohort, where a level of 3.6 pmol/L was recorded in men with PCa remission after ADT, or 4.9 pmol/L among those with stable disease or 6.4 pmol/L in those with progressive prostate cancer while control men without PCa recorded a mean of 3.8 ± 3.2 pmol/L^16^. Although our cohort was clinically not considered as with bone metastasis, the marginally higher parathyroid hormone levels recorded in our cohort shed doubt on that. Decline in parathyroid hormone levels with ADT has been previously recorded^17^. However, the current study showed no variation in parathyroid hormone levels between men on various status of ADT and PCa controls not receiving ADT. Meanwhile, the vitamin D levels recorded by our group (24.1–32.6 ng/mL or 60–81 pmol/L) is similar to the levels recorded in the Australian study reported above^16^.

The results from the cross-sectional analysis showed significantly higher sclerostin levels in men receiving acute ADT and chronic ADT compared to PCa controls. This is consistent with the cross-sectional study examining serum sclerostin levels in ADT-treated PCa patients, PCa patients without ADT, and healthy controls, where ADT-treated PCa patients had higher levels of sclerostin than the other two groups^18^. In our cross-sectional analysis, we observed that an increase in sclerostin levels began early during the initiation of ADT, and the level remained elevated during treatment. Sclerostin levels declined after the withdrawal of ADT as observed in former ADT users (Table 2). Our study is the first longitudinal study examining the effects of androgen suppression on sclerostin levels.

A recent prospective study showed that postmenopausal women with hormone receptor positive early breast cancer treated with aromatase inhibitors had an increase in serum sclerostin levels after 24 months of treatment^19^. However, in the present study we did not observe a significant change in sclerostin levels in acute or chronic ADT users after 6 months. This may be due to the relatively short follow-up period. A non-significant reduction in sclerostin levels was observed in the small group of acute ADT users after 6 months. This reduction could be due to confounding effects that down-regulate sclerostin levels, including lifestyle factors and seasonal changes. It has been shown that serum sclerostin is influenced by seasonal variation, in which the levels were 40% higher in winter than summer^20^. However, we did not observe a significant difference in serum sclerostin levels between patients who provided blood samples during fall/winter and spring/summer in the entire cohort, or in the subgroups (acute and chronic ADT user, former ADT users, and PCa controls) (data not shown).

Both cross-sectional and longitudinal studies have previously shown that higher levels of physical activity are associated with lower sclerostin levels^21,22^, while one study has shown an initial increase within the first hour during exercise^23^. In a longitudinal study on premenopausal women, eight weeks of a physical activity training programme resulted in a decrease in serum sclerostin levels^21^.

Serum sclerostin levels are higher in postmenopausal women than in premenopausal women, and it has been demonstrated that serum estradiol levels are inversely correlated with serum sclerostin^24,25^. We observed an inverse correlation between serum sclerostin and estradiol in the entire cohort, but not in the subgroup analysis. This could be due to the low power in stratified analyses. In addition, we also observed a negative correlation between serum testosterone and sclerostin in the entire cohort, former ADT users and PCa controls. However, there was no such correlation between serum sclerostin and testosterone or estradiol levels in acute plus chronic ADT users. This implies that inverse sclerostin testosterone correlation is lost at very low testosterone levels subsequent to castration. A wide range of sclerostin levels observed in ADT users with narrow testosterone ranges suggested a possible influence of genetic and other factors in circulating sclerostin levels. Consistent with our finding, Garcia-Fontana et al. also observed that serum sclerostin levels were inversely related to testosterone levels in PCa patients. However, these authors did not observe a significant correlation between serum sclerostin and estradiol^18^. A negative correlation between sclerostin and testosterone levels was also found in men with idiopathic osteoporosis^26^.

Morales-Santana et al.^27^ suggests that the dose relationship between estradiol levels and sclerostin production, to be an inverted U shape, with maximal sclerostin levels at the second quartile of estradiol, and the lower and higher extremes of estradiol associated with the lowest sclerostin levels. We therefore assessed the variation between estradiol and sclerostin and testosterone and sclerostin in a quartile basis for the pooled baseline data (Supplementary Figures 1 and 2). We did not observe a U shape between either estradiol and sclerostin or with testosterone and sclerostin. Instead, we observed that those within the lowest quartile of estradiol had significantly higher sclerostin level compared to those within the highest estradiol quartile. Similarly, those within the lowest testosterone quartile had significantly higher sclerostin level compared to those within the highest testosterone quartile.

The present study demonstrated that baseline serum sclerostin levels correlated positively with lumbar spine BMD in ADT users and PCa controls. This is consistent with a number of observational studies. In a cross-sectional study of 450 African American men and women with type 2 diabetes, Register et al. also reported a significantly positive correlation between circulating sclerostin levels and BMD of the lumbar spine in both genders^28^. These authors report a sclerostin level of 1217 ± 447 pg/mL in men. However, according to Wang et al. 2018 serum sclerostin levels were found to be independently associated with the presence of osteopenia and osteoporosis in type 2 diabetes patients^29^. Another cross-sectional study involving 161 healthy adults aged 19–64 years (mean age 46.0 ± 8.6 years for men and 36.7 ± 9.6 years for premenopausal women) also reported that serum sclerostin levels were positively correlated with BMD of the lumbar spine and left hip^22^. In a small study of 37 haemodialysis patients with BMD measurements, Cejka et al. reported a positive association between serum sclerostin levels and BMD of the lumbar spine, femoral neck, and distal radius^30^. These authors record a sclerostin level of 1257 ± 1032 pg/mL among haemodialysis patients and 415 ± 227 pg/mL among controls. Our PCa cohort recorded a much higher sclerostin level ranging from 2309.84 (1516.16–4887.19) to 3099.91 (2508.15–7692.70) compared to the levels reported in type 2 diabetes patients^28^ and haemodialysis patients^30^ mentioned above. A significant positive correlation between serum sclerostin and BMD of the lumbar spine was also observed in postmenopausal breast cancer patients treated with aromatase inhibitors, where sclerostin levels reportedly increased 46% due to treatment^19^. Given that osteocytes are a major source of sclerostin, the observed positive correlation between sclerostin and BMD could reflect a higher bone mass associated with a higher rate of osteocyte differentiation from osteoblasts, and this in turn is associated with higher sclerostin levels. Ma et al.^31^ have shown an association between serum sclerostin levels and vertebral bone marrow fat in older men. These authors have further shown a positive association between sclerostin and cortical and trabecular total hip BMD indicative of marrow adipogenesis and osteocyte function^31^. On the contrary, in the cross-sectional study by Garcia-Fontana et al., the authors did not observe correlations among sclerostin levels, BMD and bone turnover markers in PCa patients with or without ADT or healthy controls^18^. The consensus from the majority of these studies indicates that serum sclerostin may act as a predictor of BMD and thus consider this marker to be useful in identifying PCa patients at risk for osteoporosis. However, a recent review by Kim et al.^32^ records that sclerostin level cannot be considered as a predictor for osteoporotic fractures as contradictory reporting is available in scientific literature^32^. We have discussed below further possible outcomes of increased sclerostin levels on bone metabolism.

As the Wnt signaling pathway inhibition by sclerostin should, in theory, decrease differentiation, proliferation and survival of osteoblasts and thus reduce BMD, the positive correlation between sclerostin and BMD in acute and chronic ADT users in this, as well as other cancer-related studies, need an explanation. It is known that physical or hormonal stimuli are necessary to recruit mono-nuclear pre-osteoclasts to the bone remodelling sites, where they are fused to make multi-nucleated osteoclasts required for the regulatory resorption of organic and mineral bone components^33^. Therefore, the lack of hormonal stimuli due to ADT could have repercussions in the bone remodelling process, with possible retention of excessive organic and mineral bone components. This could be a suggestive reason for the increased BMD despite an increase in sclerostin production observed in those managed with acute and chronic ADT. However, this feature of increased BMD with increased sclerostin was evident also in our PCa controls that had no androgen suppression except for their age-based natural androgen decline. This means that in men with unresolved PCa whether being treated with ADT or not, a positive correlation between BMD and sclerostin exists. It is known that ADT increases the risk of bone fracture^3^, possibly due to the weakened bone architecture due to poor bone remodelling. It is important to remember that, the bone matrix consisting of collagenous and non-collagenous components are also important factors in fracture resistance alongside the cellular bone mass^34^. Strength of bone depends both on the bone quality, which in turn is dependent on bone architecture, mineralization, quality of the collagen and mineralisation of the bone matrix; and density of the bone^35^. Reduced sex hormone levels with advancing age of these patients may also lead to a general decline in BMD. We have previously recorded the highest level of testosterone among the PCa non-ADT controls compared to the other three groups mentioned in the current study^3^. We observed an inverse correlation between testosterone and sclerostin in non-ADT PCa controls as well as the former ADT group at baseline. Increased sclerostin, providing a direct or indirect stimulus to increase extracellular matrix secretion, could be a possibility among men with PCa regardless of ADT, thus increasing BMD. However, future studies are required to prove such functions of sclerostin. A systematic review indicated that the use of positron emission tomography (PET) with the 68 Ga-labelled prostate-specific membrane antigen (68 Ga-PSMA) test can achieve minimal residual disease detection rates ranging from 11 to 75% at serum PSA levels below 0.5 ng/mL^36^. Interestingly, in our study the former ADT group, possibly with suppressed tumor load including that of the possible bone invaded component, showed the expected inverse correlation between sclerostin and BMD, supporting our interpretation of irregular increase in BMD with increasing sclerostin could be a precautionary attempt to avoid bone invasion by tumor cells. It has been reported that there is increased production of dense, misaligned and disorganised collagen with cancer bone metastasis including in PCa^37^ and melanoma^38,39^. Therefore, there is a possibility that increased BMD recorded in men with non-ADT managed PCa as well as in the acute and former ADT group, could be harbouring micro-metastasis (minimal residual disease). Increased, but possibly irregular BMD, could be a means of resisting invading cancer cells. Although, these men were recruited in a clinically non-metastatic stage, it was not possible to exclude those men with minimal residual disease from our study. Under these circumstances, the increased BMD in PCa patients might lead to an imprecise understanding of their true bone strength unless the sclerostin marker level is known. If both are increasing together, it is a possibility that there is an irregular bone formation taking place. Former ADT users showed a significant decline in BMD with increasing sclerostin levels 6 month after study initiation, which strengthens our assumption that suppressing micro-metastasis due to previous ADT and regaining of the sex hormonal function after ADT being stopped could initiate normal bone remodelling with regular osteoclast function.

In the present study, serum sclerostin levels were positively associated with age in the entire patient cohort and in patients receiving ADT. A large study comprising 318 men and 362 women reported that serum sclerostin levels increased with age in both men and women. These authors suggested that this may be due to the increased sclerostin production by individual osteocytes which is associated with aging, but they could not exclude the possibility of reduced clearance of the protein with increasing age^40^. In the study carried out by Garcia-Fontana et al. a positive correlation with age was observed in healthy controls but not in PCa patients without ADT and ADT-treated patients^18^. Similar to the latter study, we also did not observe a positive correlation between sclerostin and age in PCa controls.

We also investigated whether ADT had an effect on serum OPG levels. The results from the cross-sectional analysis showed that men receiving chronic ADT and former ADT users had significantly higher OPG levels than PCa controls. Similarly, in a cross-sectional study consisting of 91 PCa patients (41 without ADT and 49 with ADT), serum OPG levels were higher in ADT-treated patients than patients on no ADT. However, the difference was not statistically significant^41^. In the present study, the cross-sectional analysis indicated that there is a delayed elevation in serum OPG after the initiation of ADT that persisted even after the withdrawal of ADT. The increased level of OPG observed in patients on chronic ADT appears to reflect a protective mechanism to compensate for the increased bone resorption observed in ADT users. This is supported by the proposal that increased OPG levels in ADT users may represent a homeostatic response, in an attempt to reverse bone loss. Indeed, we observed a significant increase in lumbar spine and femoral neck BMD in former ADT users after 6 months^42^. Meanwhile, Takayama et al.^43^ have recorded the benefits of OPG in castration-related bone metastasis in a castration-insensitive mouse prostate cancer model^43^. A systematic review and meta-analysis indicate that serum OPG levels are higher in PCa patients with bone metastasis, compared to PCa patients without bone metastasis and healthy controls, while the latter two groups showed no difference in OPG levels^44^. Therefore, a possibility also exists that the elevated OPG levels in our men who received chronic ADT and former ADT may have had undetected bone metastasis. However, this interpretation should be considered cautiously as the OPG levels are shown to decline with increased number of bone lesions in breast cancer^45^. The authors record a median of around 150 pg/mL in breast cancer cases without bone metastasis, compared to around 70 pg/mL in those with metastasis. Compared to the above OPG levels recorded in breast cancer patients, our PCa patients recorded higher levels ranging from 329 to 426 pg/mL.

In the cross-sectional study by Varsavsky et al., the authors reported that serum OPG levels were positively correlated with levels of total testosterone and bioavailable testosterone in patients without ADT. In men treated with ADT, serum OPG levels were positively correlated with the levels of total estradiol, bioavailable estradiol, and free estradiols^41^. However, we did not observe significant correlations between estradiol or testosterone with OPG in patients treated with ADT or PCa controls. We observed a positive correlation between serum OPG and age in patients receiving ADT and former ADT users. This is consistent with previous studies in healthy individuals and PCa patients^41,46^.

The increased OPG levels with age could represent a compensatory response to the increased osteoclastic resorption which also increases with age. In the present study, we observed a positive association between serum OPG and BMD of the lumbar spine only in PCa controls. In a cross-sectional analysis involving 618 community-dwelling adults, the authors also observed a positive association between serum OPG levels and lumbar spine BMD in older men^47^. On the contrary, Varsavsky et al. did not observe any correlation between serum OPG and BMD in PCa patients^41^.

Studies suggest that DDK-1 has a role in promoting tumour growth, PCa progression, and bone metastasis^48,49^. Therefore, the positive correlation observed in our study between DDK-1 and testosterone could be due to increased tumour load. We did not observe a significant difference in serum DKK-1 between the four groups. However, in our longitudinal analysis, we observed a significant reduction in serum DKK-1 in chronic ADT users, former ADT users, and PCa controls. The decrease of DDK-1 with ADT treatment may be due to reduction of tumour load. However, the reasoning behind reduction of DKK-1 in PCa controls after 6 months is unclear.

Meanwhile, in the general populations, increases in OPG have been shown to increase cardiovascular and diabetes risk^50,51^. It is suggested that DKK-1 also promotes inflammation and endothelial cell injury associated with subsequent formation and destabilization of atherosclerotic lesions^52,53^. A recent study has shown that in a cohort consisting of 70% men undergone coronary angiography, with a median sclerostin level of 133 pg/mL, those in the > median level of sclerostin were at increased risk of major cardiovascular events and death at 9-year follow-up^54^. The current cohort with acute- and chronic-ADT recording around 3000 ng/mL sclerostin and former-ADT group as well as the non-ADT PCa controls recording between 2300 and 2600 ng/mL sclerostin, could be under extreme risk of cardiovascular events. Therefore, ADT-related increases in OPG, DKK-1 and sclerostin and the general increase in sclerostin even in the non-ADT PCa controls is a concern. There are several limitations in the present study. First, the sample size was small, especially in the acute ADT group. This might lead to missing statistically significant changes in bone markers and BMD. Due to the small sample size, we do not have enough power to perform multiple comparison tests or adjustments. The second limitation is the relatively short follow-up time. Thirdly, the levels of bone markers were measured only at two time points (baseline and 6 months). The fourth limitation is the absence of a control group with no PCa. Sclerostin level is negatively associated with physical activity. Unfortunately, we could not adjust our results based on the physical activity level, and this too adds to study limitations.

## Conclusions

Serum levels of sclerostin were higher in acute and chronic ADT users and levels returned to the “normal range” identified as that measured in the PCa controls when treatment was withdrawn. Serum OPG seemed to have a delayed response and was significantly elevated in chronic and former ADT users. The inverse relationships observed between testosterone and sclerostin indicated that androgen plays an important role in regulating synthesis of sclerostin and bone metabolism in PCa patients. However, among acute plus chronic ADT users, a significant inverse correlation of sclerostin levels with serum testosterone was not observed. This may be due to the narrow testosterone range recorded at this treatment stage.

Sclerostin levels showed a significant positive correlation with BMD among acute and chronic ADT users at baseline and PCa controls at both baseline and at 6 months follow-up. Our data may suggest the importance of close monitoring of PCa patients that record increasing levels of sclerostin accompanying increasing levels of BMD for possible minimal residual disease. Our data also suggest that sclerostin, DKK-1 and OPG may not be surrogates for BMD among PCa patients being treated with and without ADT. Future studies with longer follow-up may provide more information regarding the impact of sclerostin on BMD in men receiving ADT. Furthermore, it will be useful to prospectively monitor these men to record any clinical manifestation of cancer metastasis and align such information with bone marker levels currently recorded. For similar future studies it will be beneficial to monitor minimal residual disease with techniques such as PET 68 Ga-PSMA. Additionally, in the future, it is worth assessing whether the well-known osteoblastic PCa metastasis^55^ leading to dense misaligned and disorganised collagen is a resistance mechanism to counteract invading cancer cells and associated with the bone marker sclerostin.

## Materials and methods

The present study is a secondary analysis from a pilot study which examined the effect of ADT on BMD and bone turnover markers in a PCa cohort in New Zealand^42^. The participants were 88 men diagnosed with PCa without known bone metastases. They had received or were receiving various treatment/management options such as ADT (GnRH agonist alone or in combination with anti-androgen), radiation therapy, radical prostatectomy, and active surveillance. The participants were categorized into four groups: (1) patients who were treated with GnRH agonists for 6 months or less at study entry (acute ADT users), (2) patients who were treated with GnRH agonists for more than 6 months at study entry (chronic ADT users), (3) patients who had previously been treated with GnRH agonists but were no longer receiving treatment or patients on intermittent ADT at study entry, and (4) patients who had not received ADT^42^.

They were recruited between October 2014 and September 2015, and were diagnosed and/or treated in Auckland and Waikato^42^. Patients aged 50 years or older and without known bone metastases were eligible to participate in the study. Participants were excluded if they had any disease that may affect their bone health (e.g. Paget’s disease, rheumatoid arthritis, hyperthyroidism, hyperparathyroidism, severe hepatic disease, or renal failure) or were using medication that may cause poor bone health, or had been treated with anti-osteoporotic therapy within the last 12 months (e.g. bisphosphonate, denosumab, estrogen receptor modulator, calcitonin, parathyroid hormone, or glucocorticoid).

### Blood collections and processing

Blood samples were collected between 8:30 a.m. and 12:00 noon after overnight fasting. At baseline and at 6 months, blood samples were collected in two 5 mL serum separation vacutainer (SST) tubes containing clot activator and serum separator gel from Becton Dickinson.

The blood collected in SST tubes was kept at room temperature for at least 30 min, to allow clotting, and then centrifuged at 1000×*g* for 15 min at room temperature to separate the serum. The serum samples were aliquoted into 1.8 mL cryotubes and stored at − 80 °C until required for assessments.

### Measurements

BMD of the L2–L4 lumbar spine, femoral neck, ultradistal forearm, one-third distal radius, and total body were measured at baseline and 6 months by DXA, using iDXA (GE-Lunar, Madison, WI) (for patients from the Auckland region), Discovery W (Hologic Inc, Bedford*,* MA, USA) (for patient from Gisborne), and XR-800 or XR-600 densitometer (Norland*,* Cooper Surgical Company, USA) (for patients from the Waikato region)^42^.

Testosterone, estradiol (E2) and parathyroid hormone (PTH) were measured by automated immunoassays on the Roche Cobas e411 (Hitachi High-Technologies Corporation, Tokyo, Japan) at the Liggins Institute, the University of Auckland. Intra-assay coefficients of variation (CVs) were 2.62% for testosterone, 2.45% for estradiol and 2.32% for PTH.

Serum sclerostin, DKK-1, and OPG were quantitatively measured using MILLIPLEX MAP human bone panel (EMD Millipore, Billerica, MA, USA), according to the manufacturer’s instructions. Measurements were performed using Luminex technology at the Auckland Cancer Society Research Centre, the University of Auckland. Intra-assay and inter-assay CVs were 6.3% and 13.7% for serum sclerostin, 5.8% and 13.7% for DKK-1, and 4.5% and 13.1% for OPG.

### Statistical analysis

The levels of bone markers (Sclerostin, DKK-1, OPG) and sex steroids were compared between acute and chronic ADT users, former ADT users, and PCa controls.

All parameters were expressed as median and 95% confidence interval (95% CI), as most of the data deviated from a normal distribution. Serum testosterone and estradiol were normally distributed in acute ADT users, but medians were reported for easier comparison. A Kruskal–Wallis test was used to analyse the differences among the groups and if significant, this test was followed by a Wilcoxon two-sample test of each pair of groups.

The baseline and 6-month data are imbalanced (the number of observations at baseline and 6 months were unequal, and the dataset consist of extreme values and outliers) Therefore, the LS mean values with corresponding standard error (SE) of the means, from the generalized linear model, were used to assess the percentage change in bone markers from baseline to 6 months. As the LS mean method standardised the data, within each group, changes from baseline for all parameters were analysed using a paired Student’s t-test. As the percentage change between the groups are not normally distributed for each parameter, the Kruskal–Wallis test was used to analyse the differences across the groups and if significant, this was followed by the Wilcoxon two-sample test for each pair of groups. The correlation between variables of interest were assessed in ADT users and PCa controls using the Spearman correlation. Due to the small sample size of the acute ADT users, acute and chronic ADT users were considered as a combined group, while former ADT users were considered separately. A *p* value of < 0.05 was considered significant. All statistical analyses were carried out using SAS (v9.4 SAS Institute, Cary, NC, USA).

### Ethics approval

This study received ethical approval from the Northern Y Regional Ethics Committee (Ethics ref: NTY/05/06/037). All study protocols were carried out in accordance with relevant guidelines and regulations of the NTY Ethics Committee.

### Informed consent

All of the participants provided written informed consent to participate in this study.

## Supplementary Information

Supplementary Figures.

## References

[1] Lepor H, Shore ND (2012). LHRH agonists for the treatment of prostate cancer: 2012. Rev. Urol..

[2] Sharifi N, Gulley JL, Dahut WL (2005). Androgen deprivation therapy for prostate cancer. JAMA.

[3] Wang A, Obertova Z, Brown C, Karunasinghe N, Bishop K, Ferguson L, Lawrenson R (2015). Risk of fracture in men with prostate cancer on androgen deprivation therapy: A population-based cohort study in New Zealand. BMC Cancer.

[4] Boyce BF, Rosenberg E, de Papp AE, Duong LT (2012). The osteoclast, bone remodelling and treatment of metabolic bone disease. Eur. J. Clin. Invest..

[5] Bellido T (2014). Osteocyte-driven bone remodeling. Calcif. Tissue Int..

[6] Xiong J, Onal M, Jilka RL, Weinstein RS, Manolagas SC, O'Brien CA (2011). Matrix-embedded cells control osteoclast formation. Nat. Med..

[7] Nakashima T, Hayashi M, Fukunaga T, Kurata K, Oh-Hora M, Feng JQ, Bonewald LF, Kodama T, Wutz A, Wagner EF (2011). Evidence for osteocyte regulation of bone homeostasis through RANKL expression. Nat. Med..

[8] Xiong J, Piemontese M, Onal M, Campbell J, Goellner JJ, Dusevich V, Bonewald L, Manolagas SC, O'Brien CA (2015). Osteocytes, not osteoblasts or lining cells, are the main source of the RANKL required for osteoclast formation in remodeling bone. PLoS ONE [Electron. Resour.].

[9] Sharifi M, Ereifej L, Lewiecki EM (2015). Sclerostin and skeletal health. Rev. Endocr. Metab. Disord..

[10] Baron R, Kneissel M (2013). WNT signaling in bone homeostasis and disease: from human mutations to treatments. Nat. Med..

[11] Honasoge M, Rao AD, Rao SD (2014). Sclerostin: recent advances and clinical implications. Curr. Opin. Endocrinol. Diabetes Obes..

[12] Brunkow ME, Gardner JC, Van Ness J, Paeper BW, Kovacevich BR, Proll S, Skonier JE, Zhao L, Sabo PJ, Fu Y (2001). Bone dysplasia sclerosteosis results from loss of the SOST gene product, a novel cystine knot-containing protein. Am. J. Hum. Genet..

[13] Balemans W, Patel N, Ebeling M, Van Hul E, Wuyts W, Lacza C, Dioszegi M, Dikkers FG, Hildering P, Willems PJ (2002). Identification of a 52 kb deletion downstream of the SOST gene in patients with van Buchem disease. J. Med. Genet..

[14] Ke HZ, Richards WG, Li X, Ominsky MS (2012). Sclerostin and Dickkopf-1 as therapeutic targets in bone diseases. Endocr. Rev..

[15] Berruti A, Cook R, Saad F, Buttigliero C, Lipton A, Tampellini M, Lee KA, Coleman RE, Smith MR (2012). Prognostic role of serum parathyroid hormone levels in advanced prostate cancer patients undergoing zoledronic acid administration. Oncologist.

[16] Murray RM, Grill V, Crinis N, Ho PW, Davison J, Pitt P (2001). Hypocalcemic and normocalcemic hyperparathyroidism in patients with advanced prostatic cancer. J. Clin. Endocrinol. Metab..

[17] Isahaya E, Hara N, Nishiyama T, Hoshii T, Takizawa I, Takahashi K (2010). Bone metabolic disorder in patients with prostate cancer receiving androgen deprivation therapy (ADT): Impact of ADT on the growth hormone/insulin-like growth factor-1/parathyroid hormone axis. Prostate.

[18] Garcia-Fontana B, Morales-Santana S, Varsavsky M, Garcia-Martin A, Garcia-Salcedo JA, Reyes-Garcia R, Munoz-Torres M (2014). Sclerostin serum levels in prostate cancer patients and their relationship with sex steroids. Osteoporos. Int..

[19] Kyvernitakis I, Rachner TD, Urbschat A, Hars O, Hofbauer LC, Hadji P (2014). Effect of aromatase inhibition on serum levels of sclerostin and dickkopf-1, bone turnover markers and bone mineral density in women with breast cancer. J. Cancer Res. Clin. Oncol..

[20] Dawson-Hughes B, Harris SS, Ceglia L, Palermo NJ (2014). Serum sclerostin levels vary with season. J. Clin. Endocrinol. Metab..

[21] Ardawi MS, Rouzi AA, Qari MH (2012). Physical activity in relation to serum sclerostin, insulin-like growth factor-1, and bone turnover markers in healthy premenopausal women: A cross-sectional and a longitudinal study. J. Clin. Endocrinol. Metab..

[22] Amrein K, Amrein S, Drexler C, Dimai HP, Dobnig H, Pfeifer K, Tomaschitz A, Pieber TR, Fahrleitner-Pammer A (2012). Sclerostin and its association with physical activity, age, gender, body composition, and bone mineral content in healthy adults. J. Clin. Endocrinol. Metab..

[23] Kouvelioti R, Kurgan N, Falk B, Ward WE, Josse AR, Klentrou P (2018). Response of sclerostin and bone turnover markers to high intensity interval exercise in young women: Does impact matter?. Biomed. Res. Int..

[24] Mirza FS, Padhi ID, Raisz LG, Lorenzo JA (2010). Serum sclerostin levels negatively correlate with parathyroid hormone levels and free estrogen index in postmenopausal women. J. Clin. Endocrinol. Metab..

[25] Modder UI, Clowes JA, Hoey K, Peterson JM, McCready L, Oursler MJ, Riggs BL, Khosla S (2011). Regulation of circulating sclerostin levels by sex steroids in women and in men. J. Bone Miner. Res..

[26] Lapauw B, Vandewalle S, Taes Y, Goemaere S, Zmierczak H, Collette J, Kaufman JM (2013). Serum sclerostin levels in men with idiopathic osteoporosis. Eur. J. Endocrinol..

[27] Morales-Santana S, Diez-Perez A, Olmos JM, Nogues X, Sosa M, Diaz-Curiel M, Perez-Castrillon JL, Perez-Cano R, Torrijos A, Jodar E (2015). Circulating sclerostin and estradiol levels are associated with inadequate response to bisphosphonates in postmenopausal women with osteoporosis. Maturitas.

[28] Register TC, Hruska KA, Divers J, Bowden DW, Palmer ND, Carr JJ, Wagenknecht LE, Hightower RC, Xu J, Smith SC (2014). Sclerostin is positively associated with bone mineral density in men and women and negatively associated with carotid calcified atherosclerotic plaque in men from the African American-Diabetes Heart Study. J. Clin. Endocrinol. Metab..

[29] Wang N, Xue P, Wu X, Ma J, Wang Y, Li Y (2018). Role of sclerostin and dkk1 in bone remodeling in type 2 diabetic patients. Endocr. Res..

[30] Cejka D, Jager-Lansky A, Kieweg H, Weber M, Bieglmayer C, Haider DG, Diarra D, Patsch JM, Kainberger F, Bohle B, Haas M (2012). Sclerostin serum levels correlate positively with bone mineral density and microarchitecture in haemodialysis patients. Nephrol. Dial. Transplant..

[31] Ma YH, Schwartz AV, Sigurdsson S, Hue TF, Lang TF, Harris TB, Rosen CJ, Vittinghoff E, Eiriksdottir G, Hauksdottir AM (2014). Circulating sclerostin associated with vertebral bone marrow fat in older men but not women. J. Clin. Endocrinol. Metab..

[32] Kim BJ, Lee SH, Koh JM (2020). Potential biomarkers to improve the prediction of osteoporotic fractures. Endocrinol. Metab. (Seoul).

[33] Wittkowske C, Reilly GC, Lacroix D, Perrault CM (2016). In vitro bone cell models: Impact of fluid shear stress on bone formation. Front. Bioeng. Biotechnol..

[34] Sroga GE, Vashishth D (2012). Effects of bone matrix proteins on fracture and fragility in osteoporosis. Curr. Osteoporos. Rep..

[35] Bienz M, Saad F (2015). Androgen-deprivation therapy and bone loss in prostate cancer patients: A clinical review. Bonekey Rep..

[36] Eissa A, Elsherbiny A, Coelho RF, Rassweiler J, Davis JW, Porpiglia F, Patel VR, Prandini N, Micali S, Sighinolfi MC (2018). The role of 68Ga-PSMA PET/CT scan in biochemical recurrence after primary treatment for prostate cancer: a systematic review of the literature. Minerva Urol. Nefrol..

[37] Sekita A, Matsugaki A, Nakano T (2017). Disruption of collagen/apatite alignment impairs bone mechanical function in osteoblastic metastasis induced by prostate cancer. Bone.

[38] Sekita A, Matsugaki A, Ishimoto T, Nakano T (2017). Synchronous disruption of anisotropic arrangement of the osteocyte network and collagen/apatite in melanoma bone metastasis. J. Struct. Biol..

[39] Kolb AD, Bussard KM (2019). The bone extracellular matrix as an ideal milieu for cancer cell metastases. Cancers (Basel).

[40] Modder UI, Hoey KA, Amin S, McCready LK, Achenbach SJ, Riggs BL, Melton LJ, Khosla S (2011). Relation of age, gender, and bone mass to circulating sclerostin levels in women and men. J. Bone Miner. Res..

[41] Varsavsky M, Reyes-Garcia R, Aviles Perez MD, Gonzalez Ramirez AR, Mijan JL, Munoz-Torres M (2012). Serum osteoprotegerin and sex steroid levels in patients with prostate cancer. J. Androl..

[42] Wang A, Karunasinghe N, Plank L, Zhu S, Osborne S, Bishop K, Brown C, Schwass T, Masters J, Holmes M (2017). Effect of androgen deprivation therapy on bone mineral density in a prostate cancer cohort in New Zealand: A pilot study. Clin. Med. Insights Oncol..

[43] Takayama K, Inoue T, Narita S, Maita S, Huang M, Numakura K, Tsuruta H, Saito M, Maeno A, Satoh S (2017). Inhibition of the RANK/RANKL signaling with osteoprotegerin prevents castration-induced acceleration of bone metastasis in castration-insensitive prostate cancer. Cancer Lett.

[44] Zang L, Ma M, Hu J, Qiu H, Huang B, Chu T (2015). The effects of lung and prostate cancer bone metastasis on serum osteoprotegerin levels: A meta-analysis. Sci. Rep..

[45] Elfar GA, Ebrahim MA, Elsherbiny NM, Eissa LA (2017). Validity of osteoprotegerin and receptor activator of NF-kappaB ligand for the detection of bone metastasis in breast cancer. Oncol. Res..

[46] Indridason OS, Franzson L, Sigurdsson G (2005). Serum osteoprotegerin and its relationship with bone mineral density and markers of bone turnover. Osteoporos. Int..

[47] Stern A, Laughlin GA, Bergstrom J, Barrett-Connor E (2007). The sex-specific association of serum osteoprotegerin and receptor activator of nuclear factor kappaB legend with bone mineral density in older adults: the Rancho Bernardo study. Eur. J. Endocrinol..

[48] Hall CL, Zhang H, Baile S, Ljungman M, Kuhstoss S, Keller ET (2010). p21CIP-1/WAF-1 induction is required to inhibit prostate cancer growth elicited by deficient expression of the Wnt inhibitor Dickkopf-1. Cancer Res..

[49] Thudi NK, Martin CK, Murahari S, Shu ST, Lanigan LG, Werbeck JL, Keller ET, McCauley LK, Pinzone JJ, Rosol TJ (2011). Dickkopf-1 (DKK-1) stimulated prostate cancer growth and metastasis and inhibited bone formation in osteoblastic bone metastases. Prostate.

[50] Tschiderer L, Willeit J, Schett G, Kiechl S, Willeit P (2017). Osteoprotegerin concentration and risk of cardiovascular outcomes in nine general population studies: Literature-based meta-analysis involving 26,442 participants. PLoS ONE.

[51] Bjerre M, Hilden J, Winkel P, Jensen GB, Kjoller E, Sajadieh A, Kastrup J, Kolmos HJ, Larsson A, Arnlov J (2020). Serum osteoprotegerin as a long-term predictor for patients with stable coronary artery disease and its association with diabetes and statin treatment: A CLARICOR trial 10-year follow-up substudy. Atherosclerosis.

[52] Di M, Wang L, Li M, Zhang Y, Liu X, Zeng R, Wang H, Chen Y, Chen W, Zhang Y, Zhang M (2017). Dickkopf1 destabilizes atherosclerotic plaques and promotes plaque formation by inducing apoptosis of endothelial cells through activation of ER stress. Cell. Death Dis..

[53] Ueland T, Otterdal K, Lekva T, Halvorsen B, Gabrielsen A, Sandberg WJ, Paulsson-Berne G, Pedersen TM, Folkersen L, Gullestad L (2009). Dickkopf-1 enhances inflammatory interaction between platelets and endothelial cells and shows increased expression in atherosclerosis. Arterioscler. Thromb. Vasc. Biol..

[54] Kern A, Stompor T, Bil J (2021). Sclerostin and cardiovascular disease: any prognostic implications? Authors' reply. Kardiol. Pol..

[55] Lin SC, Yu-Lee LY, Lin SH (2018). Osteoblastic factors in prostate cancer bone metastasis. Curr. Osteoporos. Rep..

