# Supplementary material for: Effect of androgen deprivation therapy on serum levels of sclerostin, Dickkopf-1, and osteoprotegerin: a cross-sectional and longitudinal analysis
Source: Sci Rep. 2021 Jul 21;11:14905. doi: 10.1038/s41598-021-94090-y (PMC8295319; doi:10.1038/s41598-021-94090-y)
Supplement: Supplementary file 1 — Supplementary Figures. [file 41598_2021_94090_MOESM1_ESM.docx]

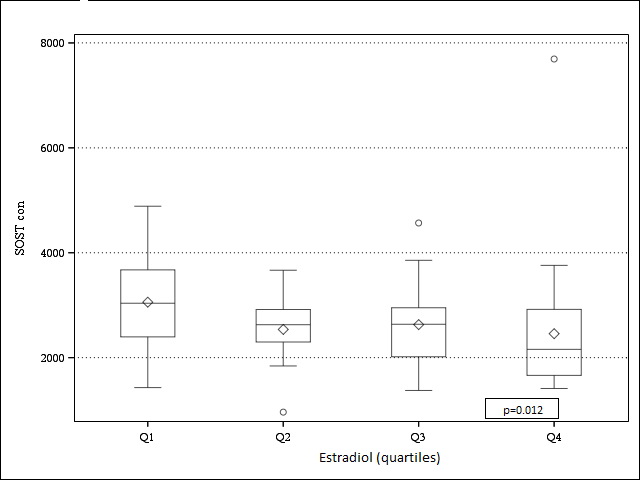


Supplementary figure 1: Comparison of sclerostin levels with estradiol quartiles in pooled baseline data.


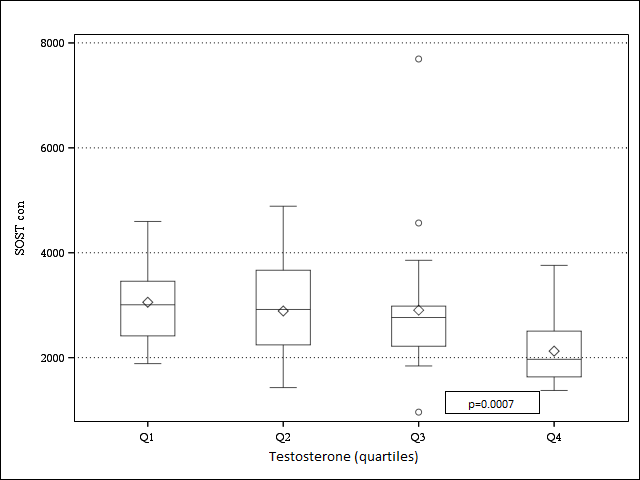


supplementary figure 2 : Comparison of sclerostin levels with testosterone quartiles in pooled baseline data.
